# Emergence and Progression of Behavioral Motor Deficits and Skeletal Muscle Atrophy across the Adult Lifespan of the Rat

**DOI:** 10.3390/biology12091177

**Published:** 2023-08-28

**Authors:** Max GrönholdtKlein, Ali Gorzi, Lingzhan Wang, Erik Edström, Eric Rullman, Mikael Altun, Brun Ulfhake

**Affiliations:** 1 Department of Neuroscience, Karolinska Institutet, 171 77 Stockholm, Sweden; maxgronholdtklein@gmail.com; 2 Department of Sport Sciences, University of Zanjan, Zanjan 45371-38791, Iran; ali_gorzi@znu.ac.ir; 3 Department of Human Anatomy, Histology and Embryology, Inner Mongolia Minzu University, Tongliao 028000, China; wlzh001@aliyun.com; 4 Department of Clinical Neuroscience, Karolinska Institutet, 171 77 Stockholm, Sweden; erik.edstrom.1@ki.se; 5 Department of Laboratory Medicine, Karolinska Institutet, 171 77 Stockholm, Sweden; eric.rullman@ki.se (E.R.); mikael.altun@ki.se (M.A.)

**Keywords:** aging, sarcopenia, dynapenia, skeletal muscle atrophy, denervation, behavior

## Abstract

**Simple Summary:**

Much of what we know about the loss of skeletal muscle strength and mass (sarcopenia) in old age comes from studies of small laboratory rodents. In humans, this condition is generally not noticeable until late middle age when it affects our mobility (clinical stage). As muscle wasting progresses with age, it poses a challenge to independent living and worsens the health of those of us already suffering from other diseases. WHO and national health authorities have identified sarcopenia as one of the major age-related diseases we must try to curb. Observations in humans show that the process underlying sarcopenia goes on for decades before we notice it (preclinical phase). In this study, we show that the same disease process is observed in rats as in humans, and that skeletal muscles exhibit a series of adaptive responses to maintain muscle function and mass during the long period that precedes clinical symptoms. When these adaptive mechanisms are exhausted, the disease progresses to a clinical stage. We conclude that the rat is a useful model to further study the triggering mechanisms of this disease and, moreover, is suitable for assessing whether interventions to treat the disease are more successful in the preclinical phase than in the clinical phase.

**Abstract:**

The facultative loss of muscle mass and function during aging (sarcopenia) poses a serious threat to our independence and health. When activities of daily living are impaired (clinical phase), it appears that the processes leading to sarcopenia have been ongoing in humans for decades (preclinical phase). Here, we examined the natural history of sarcopenia in male outbred rats to compare the occurrence of motor behavioral deficits with the degree of muscle wasting and to explore the muscle-associated processes of the preclinical and clinical phases, respectively. Selected metrics were validated in female rats. We used the soleus muscle because of its long duty cycles and its importance in postural control. Results show that gait and coordination remain intact through middle age (40–60% of median lifespan) when muscle mass is largely preserved relative to body weight. However, the muscle shows numerous signs of remodeling with a shift in myofiber-type composition toward type I. As fiber-type prevalence shifted, fiber-type clustering also increased. The number of hybrid fibers, myofibers with central nuclei, and fibers expressing embryonic myosin increased from being barely detectable to a significant number (5–10%) at late middle age. In parallel, TGFβ1, Smad3, FBXO32, and MuRF1 mRNAs increased. In early (25-month-old) and advanced (30-month-old) aging, gait and coordination deteriorate with the progressive loss of muscle mass. In late middle age and early aging due to type II atrophy (>50%) followed by type I atrophy (>50%), the number of myofibers did not correlate with this process. In advanced age, atrophy is accompanied by a decrease in SCs and βCatenin mRNA, whereas several previously upregulated transcripts were downregulated. The re-expression of embryonic myosin in myofibers and the upregulation of mRNAs encoding the γ-subunit of the nicotinic acetylcholine receptor, the neuronal cell adhesion molecule, and myogenin that begins in late middle age suggest that one mechanism driving sarcopenia is the disruption of neuromuscular connectivity. We conclude that sarcopenia in rats, as in humans, has a long preclinical phase in which muscle undergoes extensive remodeling to maintain muscle mass and function. At later time points, these adaptive mechanisms fail, and sarcopenia becomes clinically manifest.

## 1. Introduction

In middle age and beyond, the speed, strength, and mass of our muscles decrease. This facultative loss of muscle mass and function (sarcopenia) poses a serious threat to our independence and health as we age. Sarcopenia was recognized in 2002 by WHO [1] as a condition in the elderly associated with a number of diseases and is now a disease entity with the ICD-10-CM code (M62.84). Guidelines for diagnostic criteria have been compiled and revised by European, Asian, and North American working groups (the EWGSOP2 [2,3], the Asian Working Group for Sarcopenia (AWGS) [4], and the FNIH [5]) They define primary sarcopenia as a loss of skeletal muscle mass and a decrease in muscle strength (static handgrip strength) and motor performance (usually gait speed). Sarcopenia becomes a clinical condition between the ages of 60 and 70 (i.e., early aging), and later sarcopenia may be a major cause of disability [6]. However, studies suggest that muscle function already declines at the transition between young adulthood and early middle age [7,8,9,10,11,12,13], suggesting that sarcopenia may develop from processes that were initiated early and, when becoming clinically manifest, have been ongoing for decades.

Much of what we know about sarcopenia derives from studies on laboratory rodents which serve as models for the human condition (reviewed in [8,14,15,16,17,18]). Ballak et al. concluded that rats better reflect the human condition than mice [15], while mice provide a more flexible platform for genetic manipulation. Aged skeletal muscles of rodents [18,19,20,21,22,23,24,25,26,27,28,29,30,31,32,33] and humans [34,35,36,37,38,39,40,41,42] consistently show loss and atrophy of fast-twitch type II myofibers [43]. This is accompanied by a decline in satellite cells (SCs, stem cells for renewal/expansion of the myocyte muscle pool) and a decrease in SC replication rate (at least in vitro) [44,45]. Aged skeletal muscle also shows signs of inflammation, accumulation of extracellular matrix components, and infiltration of fat within and between myofibers (idem). The combined effect of these changes is seen in the loss of muscle mass and function with age. Factors thought to be responsible for these changes include the accumulation of senescent cells and their secretome in muscle, systemic changes in inflammatory and endocrine signaling, age-related changes in the cellular balance between the synthesis and degradation of proteins along with changes in nutrient balance and gut microbiota, and a sedentary lifestyle in older [laboratory] rodents and humans alike (idem and [33,46,47,48,49]). 

The vulnerability of fast-twitch type II myofibers, occurrence of iber-type grouping [17,29,40], and fragmentation and irregularities of the postsynaptic junctional membrane [50] suggest a neurogenic component in sarcopenia. This is also supported by the increasing number of fibers with both type I and type II myosin expression patterns (hybrid fibers), changes in the chemical phenotype of motor neurons [51], alterations in excitability and impulse propagation, and a loss of MUs and compensatory enlargement of the remaining MUs [15,17,52,53,54,55,56,57]. The question of whether a neurogenic component precedes, accompanies, or is a consequence of intrinsic skeletal muscle changes remains controversial [8,16,17,18,31,32,50,57,58,59,60,61,62], as a cause–effect relationship is difficult to establish because of the multitude of changes in sarcopenic muscles. 

In this study, we used outbred rats to investigate the natural history of sarcopenia, with the aim of comparing the emergence and progression of motor behavioral deficits with the degree of muscle wasting and finding processes in the muscle that precede the clinical phase of this disease. To this end, we used a battery of tests to determine the onset and progression of motor behavioral deficits across the adult lifespan, changes in muscle mass and histology, and the qPCR of a panel of mRNA transcripts based on our previous findings in rats [63,64,65] and humans [66]. We selected the soleus component of the triceps surae muscle from male SD rats for study because it undergoes long work cycles in daily life and is important for postural mechanisms [67]. In addition, a subset of measurements were also validated in young adult, middle-aged, and old female rats.

## 2. Material and Methods

### 2.1. Study Population

Although the main focus of this work is on male Sprague Dawley rats, rats of both sexes were used and delivered (Scanbur, Sollentuna, Sweden; Charles River, Dortmund, Germany) at ~8 weeks old (Table 1). In the vivarium, the rats were housed in macrolon cages (type 4, lid type 4) with aspen chips as bedding, kept 4–5 per cage with a holding room temperature of 21 ± 2 °C and 40–60% humidity, and a 12 h light–dark (LD) cycle. The rats had ad libitum access to rodent feed (R70, Lactamin) and weakly chlorinated water, refreshed weekly. Environmental enrichment was provided with plastic tubes and chew sticks. Health inventories at the facility were conducted in accordance with the FELASA recommendation, and besides Helicobacter pylori not being included in the testing panel, the animals were free of pathogens named on the FELASA exclusion list. Prior to experiments, the rats were acclimatized for approximately two weeks. Welfare checks were executed by trained technicians under the supervision of the designated veterinarian. 

Based on the outcome of behavioral testing and gross metrics, see below, a group of 18-month-old males was added and substituted for the 12-month-old male rats in the muscle histology and gene expression analyses of this study.

The use of laboratory rats and all experimental procedures were agreed upon, reviewed, and approved by the regional Laboratory Animal Ethics Council (Stockholms Djurförsöksetiska nämnd; project licenses N122/03, N122-N124/06). 

### 2.2. Behavioral Testing

Animals of both sexes were subjected to a standardized set of behavioral tests according to a previously published protocol [68] at an age of 3 months (female rats), 5 months (male rats), 12 months (female and male rats), 25 months (male rats), and at the study endpoint (30 months, male and female rats) (Table 2). Briefly, the test battery included the following:

#### 2.2.1. Open Field Activity

Explorative behavior was studied in a square area with walls (70 × 70 × 30 cm) of gray-coloured plastic. For 3 min, the movement distance, number of rearings, and number of grooming behaviors were recorded.

#### 2.2.2. Crossing a Wire Mesh Screen

A 70 cm long, 2.5 cm wire mesh screen was used to record the time to pass and number of limb placement errors.

#### 2.2.3. Beam Balance

A 2.5 cm wide wooden beam was suspended 50 cm above a soft surface. The rat was placed on the beam for a maximum of 60 s. The time on beam was recorded, and the performance was ranked according to [68]. The test was repeated three times, and the mean outcome was taken as the result of the test.

#### 2.2.4. Walking Track Analysis

The animals’ feet were stained with nontoxic acrylic paint (forepaws with red and hindpaws with black colour), and they then had to walk through an 8.5 × 42 cm transparent Plexiglas tunnel with the “home cage” as the “attractor” at the other end. The walking tracks were used to estimate (a) stride length (distance between forepaw–forepaw and hindpaw–hindpaw) and (b) gait width (distance between left and right hindpaws) as previously described (idem).

#### 2.2.5. Gait Score

We followed the protocol described in detail in [68]. Briefly, the scoring is based on the assessment of the limbs’ body-weight-bearing capacity (from 0 = walk/stand on digits; up to 3 = fail to raise the body trunk completely from the supportive surface) and if each limb shows a complete gait cycle (stance, paw-off, swing, paw-on) coordinated with the other limb(s). Scoring 0 = all phases of the gait cycle are present, and movement is coordinated with the other limbs; 3 = gait cycle phases are missing and not coordinated. Gait score 0 = no signs of dysfunction, 1 = mild or infrequent errors, 2 = regular gait errors/insufficient weight-bearing support, and 3 = severe gait dysfunction. Gait scoring was executed on all rats included in this study (Table 2).

### 2.3. Experimental Endpoint

The animals were humanely euthanized at 3-, 5-, 12-, 18-, 25-, and 30-months by an overdose of pentobarbital sodium, and during the terminal anesthesia, the components of the triceps surae muscle were quickly harvested. The fresh tissue was weighed, snap frozen in liquid-nitrogen-chilled isopentane, and stored at −80 °C until further use.

### 2.4. Histology

#### 2.4.1. Counting of Myofibers and Myonuclei with a Central Position

We followed our previously published protocol [65] (see also Supplementary Materials for details). From male rats, the soleus muscle in young adult (5-month-old), middle-aged (18-month-old), early-aged (25-month-old), and advanced-aged (30-month-old, endpoint) rats were transversely sectioned distal to muscle origin (i.e., the portion of the muscle belly that should hold all myofibers of the soleus muscle) into series of 8 or 10 μm thick sections in a cryostat and thawed onto gelatin-coated slides. The sections were stained with hematoxylin and eosin (Eosin-HTX) according to a standard protocol (see Supplementary Materials). The stained tissue sections mounted on coded slides were examined with a Nikon Optiphot (×10/0.45 dry plan-apo objective). Images were captured with a Hamamatsu C8484-05G digital camera. The photos were composited to pictures covering the whole muscle cross-section in Adobe Photoshop CSS 12.1*64 or ImageJ (version 1.45S). The pictures were opened in ImageJ, and the total number of fibers and number of fibers with central nuclei (CN) were recorded using the Cell Counter plug-in of ImageJ. Slide codes were disclosed after counting had finished.

#### 2.4.2. Immunohistochemistry

Adjacent or near adjacent 8 μm thick sections prepared in the section series described above were dried at RT for 30 min. A well was created using a DAKO pen. The tissue was then fixated in 4% formaldehyde for 10 min, then rinsed in cold tap water for 10 min, repeated three times. Epitopes were blocked with 5% normal donkey serum for one hour at RT. Primary antibodies toward embryonic (MyHCe/MyHC3; moAb, DSHB), slow (MyHCs, type I, moAb Novocastra), and fast (MyHCf, type II, moAb Novocastra) myosins, respectively, were incubated for either 1 h at 37 °C or overnight at 4 °C (see Supplementary Materials). Following incubation with the primary antibody and repeated washing, the sections were incubated with a secondary antibody for one hour at RT and kept away from exposure to light. After washing, the sections were mounted with DAPI and coverslipped. Sections were then stored at 4–8 °C before imaging.

For the counting of nuclei presumed to be satellite cells, DAPI (Vector Lab) was used as an unspecific marker of nuclei. Primary antibodies (sequential labeling for each epitope) were either directed toward Pax7 (DS Hybridoma Bank; see Supplementary Materials) and laminin (the binding site or Sigma; see Supplementary Materials) or against Pax7 and Ki67 (Novocastra; Supplementary Materials). Following repeated washing, the sections were incubated with a mixture of secondary antibodies raised in donkey against respective species of the primary antibody and processed as described above. Imaging was captured with a LSM700 confocal laser-scanning microscope (Carl Zeiss, Jena, Germany) (for details see Supplementary Materials).

### 2.5. Fiber Typing and Stereology

The images for fiber typing were acquired with a Zeiss LSM700 confocal laser-scanning microscope. Images were processed using ZEN2012 software (Zen v3.0, Zeiss) (further details in Supplementary Materials). Multipanel figures were assembled using Adobe Photoshop CSS 12.1*64 software (Adobe Systems, San Jose, CA, USA). The analyses were performed on composite microimages as described above. The person analyzing the images was blinded to the experimental group. Three adjacent or near-adjacent sections from each rat were used: one marked for slow myosin (MyHC I), another for fast myosin (MyHC II), and a third for embryonic myosin (MyHC3). By tracing across adjacent sections, myofibers were categorized as being type I (only immunoreactive (IR) to slow myosin), type II expressing fast myosin IR only, hybrid myofiber expressing both slow and fast myosin IR, and myofibers expressing the embryonic isoform of myosin (MyHC III/MyHC3). MyHC-III colocalized with either one or both slow and fast myosin isoforms, i.e., type I, type II, and hybrid fibers. ImageJ (1.45S) software was used for data collection.

Using a grid mesh cast over the section stained for slow myosin, the muscle section was divided into subregions (grid boxes of 300k pixel size each; see Supplementary Materials and Figure S1) for analysis by unbiased systematic sampling (stereology). Only fibers that did not touch the cell borders or only touched the left or the top sides of each square were systematically counted (Figure S2, green dots). A starting grid box was selected using a random number generator, and every 15th consecutive grid box was counted until a minimum of 200 fibers per muscle was registered and mapped to immunoreactivity for one or several of MyHC3, MyHC I, and MyHC II in the adjacent sections. In cases where the regional tissue quality was compromised, the counting grid was moved six squares ahead until a grid-box-enabling analysis was reached. 

The fiber-type enclosure (fiber-type grouping, fiber-type clustering; [35]) was examined for type I fibers using the same stereological approach as described above and following the protocol described in [69]. This included the assessment of the number of neighbor (n) fibers in each age group to determine the probability NP^(n+1)^ of fiber enclosure, where N is the assessed prevalence of the fiber type in each age cohort. NP^(n+1)^ is used to calculate the frequency of enclosed fibers by a random distribution based on assessed prevalence and the number of neighboring fibers (idem).

The counting of nuclei being Pax7-IR immediately adjacent to a myofiber and within the laminin boundaries (≥117 myofibers per case) was performed on single sections by multicolour imaging as described in the Supplementary Materials. We used the same method to decide on the colocalization of Pax7-IR and KI67-IR. Ki67 was used as a marker for replicating Pax7-IR profiles [70].

Further, several sections from each group used to count the number of fibers, number of myonuclear profiles, central nuclei, expression of different MyHC immunoreactivities, and fiber CSA were subjected to independent validation by a second examiner, and the results were generally in very close agreement (<5%) and never differed by more than 10% between operators.

### 2.6. RNA Extraction and qPCR

Total RNA was prepared by the Trizol method (Invitrogen, Life Technologies, Stockholm, Sweden) using a drill knife and quantified spectrophotometrically by absorbance at 260 and 280 nm with PicoDrop (Picopet01, Picodrop Ltd., Cambridge, UK). Reverse transcriptions were performed with an Applied Biosystems High-Capacity cDNA Reverse Transcription Kit as described in detail in the Supplementary Materials.

Primers were designed using the NCBI software (version June, 2008) Primer-BLAST (for nucleotide sequence and accession number, see Supplementary Materials), and the gene transcripts analyzed here were selected based on earlier studies on aging rats [63,64], the response to axonal damage and regeneration in rats [65], and early- and advanced-aged muscles in humans [66]. Amplicons by qPCR were controlled with respect to length and the number of products with a melt curve, as well as agarose gel electrophoresis using ethidium bromide as a stain. For details on the PCR reaction, see the protocol in the Supplementary Materials. CT values for each individual were then normalized (∆CT) with the endogenous control gluthathionperoxidase-1 or β-Actin [64,71]. Correction for intra-experimental efficiency was conducted using a carryover triplicate on each plate from an aliquoted cDNA pool for identical freeze–thaw cycles.

### 2.7. Surgical Intervention

Female rats were subjected to a sciatic axotomy (n = 10 and n = 10 served as controls; Table 1) under general anesthesia at the age of 3 months. All surgical procedures were carried out in an aseptic manner. An incision was made on the posterior aspect of the right thigh, and the sciatic nerve was identified just below the ramification of the posterior biceps–semitendinosus branch, where the nerve was transected with a sharp knife and ligated (to prevent outgrowth). Recovery from anesthesia took place on a heat pad at 37 °C until rats were awake and moving, usually 3–4 h. Recovery from surgery was given 4–5 days after which a behavioral examination was performed to assess the loss of function. Only animals that had lost their ability to stand/walk on their right hindlimb paws (toes/digits) were included in the study. Following 10 days of survival, the animals were euthanized, and relevant tissues were snap frozen for further PCR analysis.

### 2.8. Statistical Analyses and Models

Analyses of variance were conducted either with a Kruskal–Wallis analysis of variance (nonparametric testing) or an ANOVA (parametric testing) with post hoc pairwise testing (multiple pairwise testing according to the Steel–Dwass–Critchlow–Fligner method) or Bonferroni post hoc testing, using either the Statistica^®^ software package 6.1 (Statsoft, Tulsa, OK, USA) or the XLSTAT^®^ add-on software for MS Excel. A Cox–Mantel test was used to explore the differences in survival rate. A *p*-value of <0.05 was considered significant. Principal component analysis (PCA) was performed using R version 3.3.3 or XLSTAT. PCA was used to (1) explore the correlations between the metrics of spontaneous activity, motor and reflex functions, histological variables, and the gene expression data of the different age cohorts and to (2) visualize the impact of age and sex on motor and coordination functions (males and females) and on gene expression and histological data (only males). 

## 3. Results

### 3.1. Survival and Gross Observations

**Figure 1:**
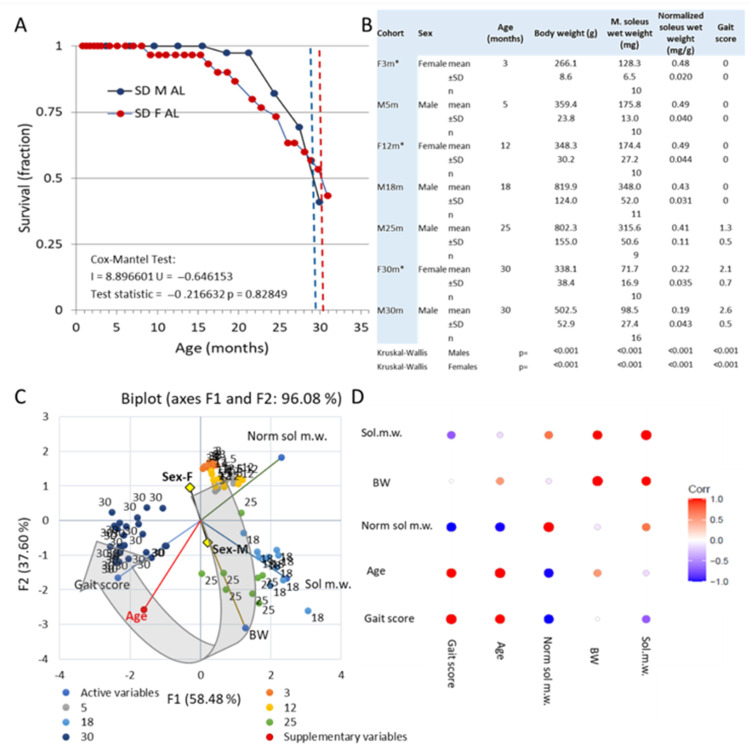
(**A**) Survival of females (red) and males (blue) for cohorts of rats maintained until they reached the median survival age (endpoint of this study). The difference between males and females was not significant (Cox–Mantel test). (**B**) Tabulation of the mean and SD of gross metrics and variance analysis per sex. (**C**) PCA analysis of gross metrics on male (5-, 18-, 25-, and 30-month-old) and female (3-, 12-, and 30-month-old) rats shows covariance with 96% of the total variance explained by the two first principal components (F1 and F2). Note that age is a supplementary variable not contributing to the factor loading on the F1 and F2 axes. As the inserted arrow indicates, male rats in late middle age (18 months old) and early aging (25 months old) separate from the cluster of young adults (male and female, 3 and 5 months old) and females in the early middle age (12 months old) mainly along F2, driven by their larger body and muscle weights and the incipient drop in normalized soleus muscle weight. In advanced age (30 months old), male and female rats separate from the other age groups mainly on the F1 axis, driven by gait deterioration and progressive muscle atrophy. Young adult males and females and early middle-aged females group tightly together. The sex-mixed group at advanced age (30 months old) forms rather dense clusters, while the observations in males at late middle age (18 months old) and in early aging (25 months old) are scattered. There is a sex difference (centroids) driven by differences in whole body size and muscle weight. (**D**) Correlation coefficients between metrics have been color coded with shades of red denoting a positive correlation, while shades of blue indicate an inverse correlation. * data replotted from [64].

The median survival age of female (~30.5 months) and male (~29.3 months) rats studied to the endpoint was not significantly different (*p* = 0.83; Figure 1A). Both female and male rats gained body weight in young adulthood and middle age (Figure 1B): female rats ~131% (12 months old), while male rats gained ~230% by 18 months of age. In parallel, there was an increase in skeletal muscle mass that matched (~135%, 12-month-old females) or nearly matched (~200%, 18-month-old males) the change in total body weight (Figure 1; see also Figure S3, Supplementary Materials). Thus, relative muscle mass, normalized m. soleus muscle mass (muscle weight/total body weight, mg/g, [29,72]) remained largely unchanged from young adulthood to middle age. In late middle age (18 months old) and early age (25 months old), there is a gradual decline in normalized muscle mass that becomes accentuated in advanced age (30 months old), resulting in a marked decrease in both sexes (Figure 1). During early aging, the loss of skeletal muscle mass is accompanied by the appearance of gait disturbances, which are seen in some but not all subjects (Figure 1B,C; see Supplementary Materials (Figure S3) for detailed boxplots with pairwise statistical tests). As aging progressed, the decrease in normalized soleus mass, gait pattern, and coordination worsened (Figure 1B,C). There was a strong correlation (r2 ≥ 0.75; *p* < 0.001) between gait score (see Materials and Methods), normalized soleus mass, and age in female and male rats across the adult lifespan (Figure 1C,D).

### 3.2. Emergence of Behavioral Motor Impairments of Aging Rats

**Figure 2:**
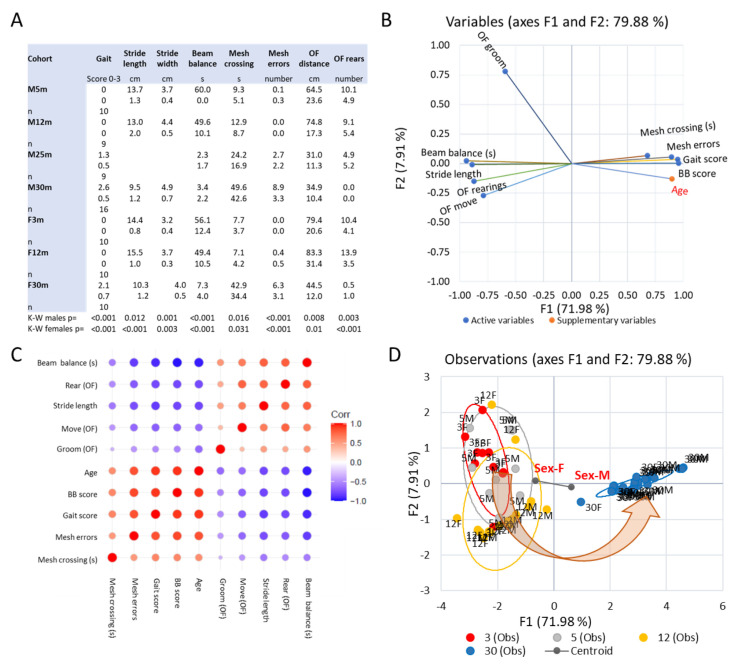
(**A**) Tabulation of behavioral test metrics (mean, SD) and sex-wise analysis of variance across age groups. (**B**) PCA analysis of activity, motor, and coordination indices in male and female age cohorts (3-, 5-, 12- and 30-month-old) shows a high degree of covariance with 80% of the total variance explained by the two first principal components (F1 and F2). Note that age is a supplementary variable not contributing to the factor loading on the F1 and F2 axes. Motor and coordination indices loaded mainly on F1, being directly or inversely mutually correlated. (**C**) Correlation coefficients between metrics in (**B**) have been color coded, with shades of red denoting a positive correlation, while shades of blue indicate an inverse correlation. (**D**) Young adult female and male rat observations overlap extensively as do the male and female observations in early middle age, while the observations on middle-aged rats of both sexes are slightly shifted away from the young adults on the F2 axis, driven by changes in the activity indices in the OF test and a shorter time on the BB. At advanced age, the observations are significantly separated from both young adult and middle-aged rats along the F1 axis because of the deterioration of motor and coordination capacities. There is a sex difference (centroids connected with a line) driven by middle- and advanced-aged female rats performing somewhat better than their male counterparts. Observations on early-aged males (25 months old) not included in the comparison of both sexes formed a group positioned in between the middle-aged and 30-month-old rats (see Supplementary Materials (Figure S4)).

We used a battery of behavioral tests [68,73] to assess changes in motor performance and coordination across the adult lifespan of male and female rats (3- and 5-, 12- and 30- month-old rats) but also changes in the level of spontaneous activity shown in the open field test (Figure 2A). As shown in Figure 2B,C, we found strong covariances between these behavioral indices and only a small difference between the sexes (Figure 2D). Observations in early-aged 25-month-old male rats were intermediate between observations in 12-month-old and 30-month-old female and male rats (see Supplementary Materials (Figure S4)).

**Figure 3:**
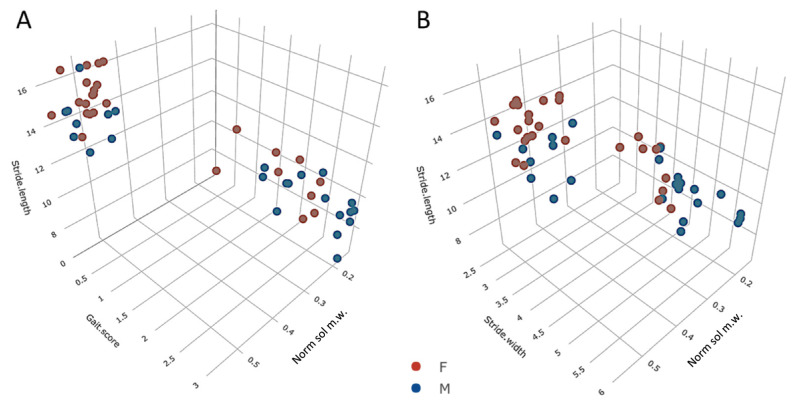
Three-axis plots of the covariation between normalized soleus mass, on the one hand, and (**A**) stride length and gait score, and (**B**) stride width and stride length, on the other. Observations for males are in blue, and females are in red. Multiple R(z/xy) for female and male rats were in (**A**) 0.89 (both sexes; *p* = 1 × 10^−7^) and in (**B**), r = 0.88 (males, *p* = 2 × 10^−7^) and r = 0.87 (females, *p* = 1 × 10^−7^).

In both sexes, all behavioral metrics showed significant changes across the adult lifespan (K-W *p* < 0.03 to *p* < 0.0001; for age-wise comparison within sexes, see Supplementary Materials (Figure S5)). Gait indices (score, stride length, stride width) and normalized soleus mass were closely correlated (proportion of variance explained > 75%; Figure 3).

#### Changes to the Soleus Muscle: The Preclinical Phase

**Figure 4:**
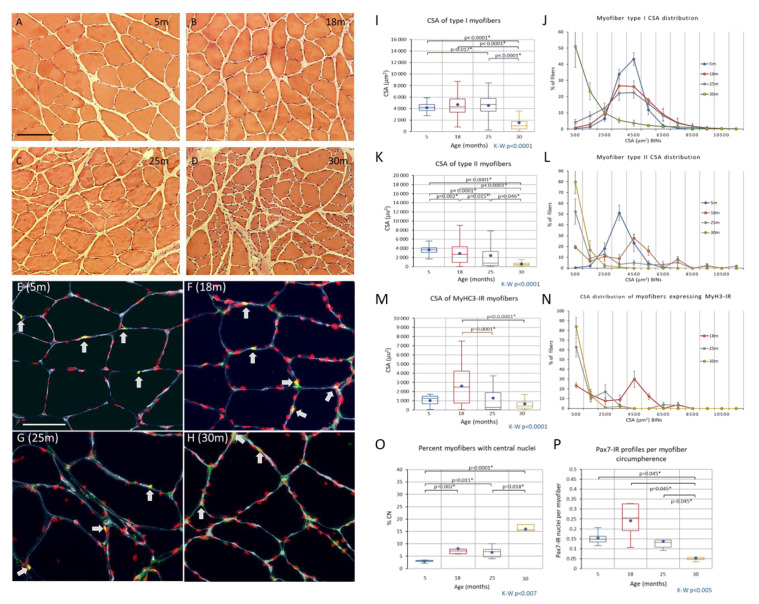
(**A**–**D**) Cross-sections of m. soleus stained with EosinHTX from young adult (**A**), middle-aged (**B**), early-aged (**C**), and advanced-aged (**D**) rats. With advancing age, the shape and size variability increases, as does the interstitial matrix space. Nuclei with a central position in the myofiber become more frequent during aging. (×10 plan-apo objective). Scale bar in (**A**) is 100 μm and applies to all panels. (**E**–**H**) Pseudo-colored three-channel confocal images with DAPI (red), Pax7-IR (green), and laminin (blue) of samples from young adult (**E**), middle-aged (**F**), early-aging (**G**) and advanced-age (**H**) rats. SC appear as yellow profiles (examples indicated by arrows) from the blending of red (DAPI) and green (Pax7-IR) light and are situated in the SC niche. Scale bar in (**A**) is 50 μm and applies to all panels. (**I**–**N**) Boxplots (**I**,**K**,**M**) of the CSA of m. soleus during aging. Age groups have been indicated on the abscissa and metrics on the ordinate in (**I**,**K**,**M**). (**J**,**L**,**N**) are plots showing the distribution of CSAs (in BINs of 1000 μm^2^) for type I—(**J**), type II—(**L**), and embryonic-myosin-expressing (**N**) myofibers. (**O**) Boxplot of the percentage of fibres having nuclei with a central position in the myofiber. (**P**) Boxplot showing the frequency of Pax7-IR nuclei per myofiber circumference for each age group. In (**O**,**P**), a Kruskal–Wallis analysis of variance indicated that each metric changed significantly during aging. Pairwise post hoc testing results have been indicated in (**O**,**P**) where they were significant (*). There is a shift in iber-type composition.

**Figure 5:**
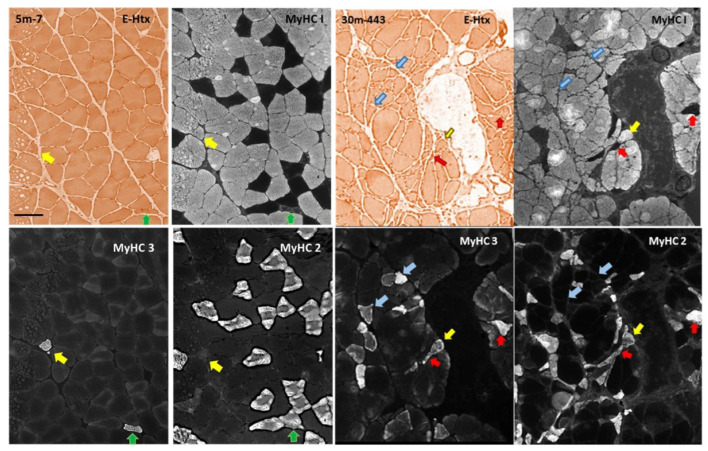
Series of near-adjacent sections from a young adult (5m-7; left four panels) and a rat at endpoint age (30m-433; four right panels) stained with Eosin-HTX, and for MyHC I-IR, yHC3-IR, and MyHC II-IR. In the panels of the young adult rat, a yellow arrow indicates a myofiber profile IR for MYHC I and MyHC3 but negative for MyHC II-IR. A green arrow points to a fine-caliber fibre IR to MyHC II and 3, and faintly positive for MyHC I. In the panels to the right, the aged rat displays examples of myofibers IR for all three myosins (yellow arrow), IR to MyHC I and MyHC3 (blue arrows), and IR for MyHC II and MyHC3 (red arrows). Also note the high degree of shape variability among aged myofibers. The full m. soleus cross-section from which the cutouts are used here to illustrate the changes imposed by aging is available in the Supplementary Materials (Figure S6). (×10 plan-apo objective), scale bar in upper left panel is 100 μm and applies to all panels.

**Figure 6:**
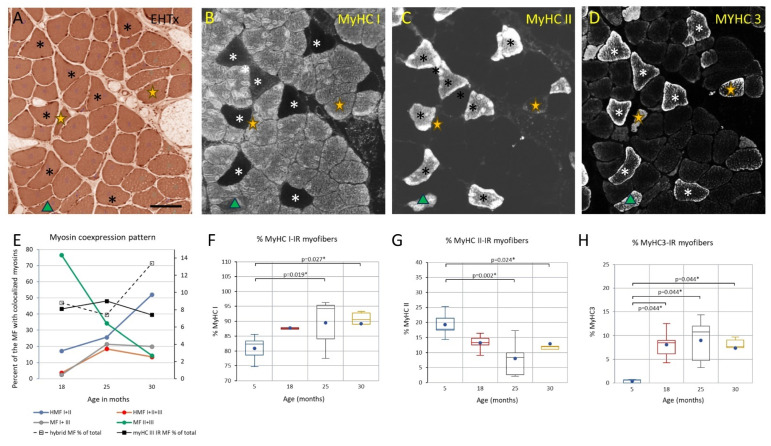
(**A**–**D**) An example showing that myofibers coexpressing type II and embryonic myosin (white * marked fibers in **A**–**D**) in the late middle-aged or early-aged rats are common and can have a well-preserved CSA. One of the fibers co-express type I and embryonic myosin but not type II myosin and has been labelled with a yellow ☆ in (**A**–**D**). (**A**–**D**) are adjacent sections stained for EHtx (**A**), type I myosin-IR (**B**), type II myosin-IR (**C**) and embryonic (MyHC3)-IR (**D**). Scale in A is 75 μm and applies to (**A**–**D**). (**E**) Plot showing the relative change between 18-, 25-, and 30-month-old rats (abscissa) in the percentage (left ordinate) of myofibers (MFs) expressing different combinations of myosins: the percentage of the total number of myofibers (ordinate to the right) in each age cohort expressing MyHC3 (black solid line) or being defined as a hybrid I/II fiber (HMF; interrupted black line). Data from young adult rats were excluded because the prevalence of such fibers was <1%. (**F**–**H**) Boxplots of the frequency of type I and type II fibers, and fibers expressing embryonic myosin during the adult age span. Age groups have been indicated on the abscissa and metrics on the ordinate in (**F**–**H**). A Kruskal–Wallis analysis of variance indicated that each metric changed significantly during aging. Pairwise post hoc testing results have been indicated where they were significant (*).

In young adulthood, the distribution of the cross-sectional area of myofibers (CSA) is fairly narrow (Figure 4A,I–N) with only a small difference (~10%) between type I and type II fibers (idem). Eighty percent of myofibers are type I, with the remainder being type II. The presence of hybrid myofibers and fibers expressing the embryonic isoform of myosin is very rare (<1%; Figure 4A,O; Figure 5 and Figure 6H). The frequency of myonuclei with a central position is also low (~3%; idem).

**Figure 7:**
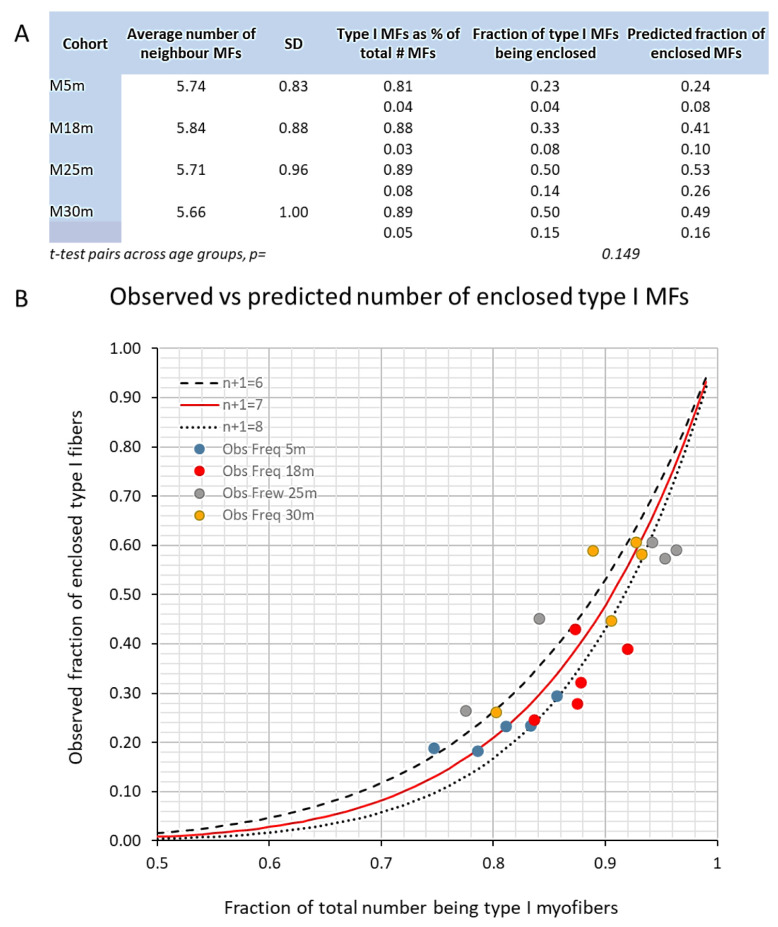
(**A**) panel indicates the mean and SD for the number of myofiber (MF) neighbors, prevalence of type I myofibers, observed number of enclosed type I myofibers, and the predicted number of enclosed type I myofibers in each age cohort. (**B**) shows the observed fraction of enclosed type I myofibers (ordinate) plotted against the prevalence of type I myofibers. The three curves (solid, interrupted, and dotted) show the expected number of enclosed myofibers by a random distribution as a function of type I prevalence (abscissa) if the number of neighbors equals 6 (interrupted line), 7 (solid red line), or 8 (dotted line), respectively (key in graph).

While muscle mass relative to body weight and motor behavior remains intact through middle age (Figure 1 and Figure 2), the soleus muscle shows a range of adaptive/regenerative changes. Compared with young adults, soleus myofibers from male rats in late middle age show an increased variability in CSA, with more large and thin myofibers (Figure 4B,I–N). The CSA of type I myofibers is maintained on average, whereas type II shows a marked decrease in CSA (idem). There is a shift in the composition of fiber types toward type I (Figure 6F,G) and an increase in fibers coexpressing multiple myosins (Figure 6). Overall, the frequency of myosin colocalization increases from <1% to approximately 9%, and at this age, the most common combination is type II and embryonic myosin (~75%), followed by fibers co-expressing type I and type II myosin (~18%) (Figure 6E). At this age, many of the myofibers coexpressing embryonic myosin and type II myosin have a CSA that is not very different from that of normal type II fibers (Figure 6A–D,G–H). The shift in fibertype composition with an increased frequency of type I myosin-IR fibers resulted in a parallel shift in the grouping of type I fibers, from 23% in adulthood to 33% in late middle age (i.e., the percentage of type I myofibers completely enclosed by fibers of the same type; Figure 7). Another sign of adaptation/regeneration at this age was the increased number of myofiberswith nuclei in a central position (~7%; Figure 4O) and the tendency toward a higher density around the myofibers (from 0.15 to 0.24) of Pax7-IR nuclei, which are presumably satellite cells (SCs) (Figure 6F).

**Figure 8:**
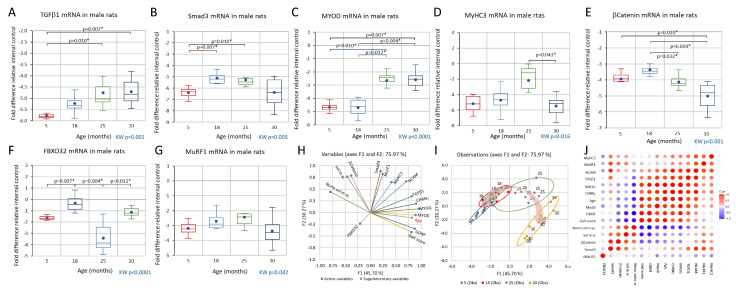
(**A**–**G**) Boxplots of TGFβ1, Smad3, FBXO32, MurF1 MYOD, βCatenin, and MyHC3 mRNA expression in m. soleus during the adult lifespan in male rats. Age groups have been indicated on the abscissa and the fold difference relative internal control on the ordinate. A Kruskal–Wallis analysis of variance indicated that each mRNA changed significantly during aging (see KW in panels). Pairwise post hoc testing results have been indicated when significant (*). (**H**) PCA analysis of gene expression across age groups of male SD rats reveal a high degree of direct or inverse covariance among the genes investigated with 76% of the total variance explained by the two first principal components. TGFβ1, CHRNγ, MYOG, MYOD, and GDNF mRNAs formed one cluster of covarying transcripts loading mainly on F1, while βCatenin, Smad3, MuRF1, and MyHC3 mRNAs formed another cluster loading mainly on F2. NCAM and FXBXO32 transcript levels loaded equally on F1 and F2. (**I**) Correlation coefficients between transcript levels in (**H**) have been color coded with shades of red denoting positive correlations, while shades of blue indicate an inverse correlation. In (**J**), the observations in each age group have been indicated, and the ovals stand for the 70% confidence limit of the respective group. Compared with young adults, middle-aged rats and rats in early aging separate gradually on both F2 and F1. The shift on F2 is driven by increased levels of βCatenin, Smad3, FBXO32, and MuRF1 transcripts. At advanced age (30 months old), these transcripts become downregulated. Across middle age and early and late aging, there is a gradual increase in abundance of the transcripts loading on F1. Note that *Age* is a supplementary variable and does not contribute to the loading of F1 or F2.

**Figure 9:**
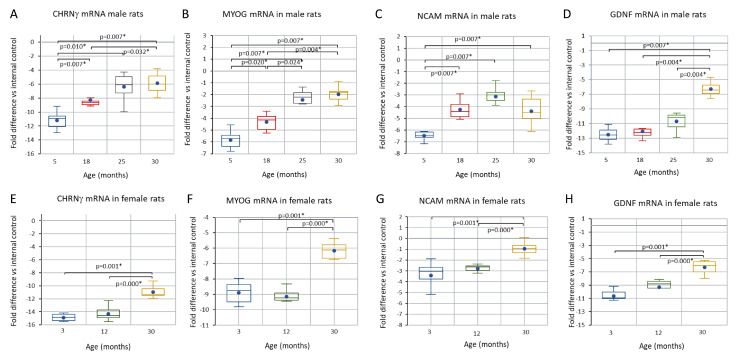
Boxplots of CHRNγ MYOG, NCAM, and GDNF mRNA abundance in m. soleus during aging in male (**A**–**D**) and female (**E**–**H**) rats. Age groups have been indicated on the abscissa and the fold difference in mRNA level relative internal control on the ordinate in (**A**–**H**). A Kruskal–Wallis analysis of variance indicated that each metric changed in a significant way during aging. Pairwise post hoc testing results have been indicated where they were significant (*).

The late middle-age changes in soleus muscle myofibers described above are associated with the upregulation of mRNA transcripts (Figure 8 and Figure 9) that encode proteins whose role in regulating myofiber contractile protein content and fiber size is well established (Figure 8A–D). The upregulation of MYOG (but not MYOD), NCAM, and CHNRγ mRNA (Figure 9A–C) with a concomitant increased expression of embryonic myosin IR suggests that transmission at neuromuscular junctions (NMJs) is impaired or disrupted.

### 3.3. Changes to the Soleus Muscle: The Clinical Phase

In early and advanced aging, as motor and coordination deficits emerge and progress (Figure 2), some of the changes observed in late middle age become more accentuated in the soleus muscle, whereas other stigmata such as myofibers expressing MyHC3 and the frequency of hybrid fibers remain at a fairly constant level from late middle age (Figure 5 and Figure 6; Figure S7 in the Supplementary Materials). Atrophy and shape change (e.g., crescent-shaped, Figure 5) of type II myofibers progresses during aging, whereas the transition from early to advanced age is characterized by a significant decrease in the CSA of type I myofibers (>50%; Figure 4 and Figure 5). The pattern of myosin co-expression also changes during early and advanced aging (Figure 6), with a significant decrease in myosin II and MyHC3 co-expression from 18 months to the endpoint, whereas hybrid fibers increase in parallel. Hybrid and myosin I-expressing fibers, which also express MyHC3, increase from 18 to 25 months of age and do not change further until the endpoint (Figure 6). These changes occur when the total proportion of myofibers IR to MyHC3 remains fairly constant, whereas the number of hybrid fibers increases by ~50% (idem, and Figure S7 in the Supplementary Materials).

The proportion of enclosed type I myofibers increases to 50% in parallel with the increased prevalence of type I myofibers (Figure 7, top panel). Observations are scattered around the lines for the expected degree of enclosure based on type prevalence and the number of fiber neighbors; the difference between the observed frequency and that expected by a random distribution was not significant across all ages (*p* = 0.159, t-test pairs) (Figure 7).

SC density per myofiber cross-sectional circumference showed a gradual decrease in density from 0.24 in 18-month-old rats to 0.05 SC per profile at the endpoint (*p* < 0.05, Figure 4). When SC density was included in the PCA, the density of Pax7-IR nuclei correlated positively with type I myofiber CSA (r = 0.623), soleus muscle weight (r = 0.70), normalized soleus mass (r = 0.54), and inversely with gait score (r = −0.77) (all correlations *p* < 0.05). The percentage of Pax7-IR nuclei also exhibiting IR Ki67 did not change significantly over the lifespan (~4% of Pax7-IR nuclei; see Supplementary Materials (Table S4)), suggesting that the replication rate per SC did not change.

### 3.4. Comparison of CHRNγ, MYOG, NCAM, and GDNF mRNAs in Aging Male and Female Rats and in the Response to Axotomy in Female Rats

The expression of CHRNγ, MYOG, NCAM, and GDNF mRNAs was also examined in young adult, middle-aged, and endpoint female rats and showed the same regulatory pattern as in males (Figure 9). In addition, CHRNγ, GDNF, and NCAM mRNA abundance was also examined in young adult female rats 10 days after complete sciatic axotomy, and the regulation of these transcripts in response to axotomy was consistent with that occurring in normal aging (see Supplementary Materials (Figure S8).

### 3.5. Trends across the Adult Lifespan

All muscle metrics except myofiber count (KW *p* = 0.295; see Supporting Information (Figure S7)) showed significant changes over the adult lifespan (KW *p* < 0.049 to *p* < 0.0001, Figure 1, Figure 4, Figure 5, Figure 6 and Figure 7; see also Supplementary Materials (Figures S6 and S7)), and there are significant covariances between several variables analyzed (Figure 1B,C, Figure 3 and Figure 8H–I). Normalized soleus mass was inversely correlated with the abundance of GDNF (r = −0.88), MYOG (r = −0.69), MYOD (r = −0.65), and CHRNγ transcripts (−0.64) across all age groups (all *p* < 0.05). Gait score showed an equally strong covariation with these transcripts (GDNF, r = 0.85; MYOG, r = 0.74; MYOD, r = 0.73; CHRNγ, r = 0.58). A similar pattern was also seen for TGFβ1 mRNA expression. In contrast, intercorrelated transcript levels of βCatenin, Smad3, and MuRF1 showed an initial increase followed by downregulation at the endpoint (Figure 8).

## 4. Discussion

### 4.1. General Comments and Limitations of This Study

Small rodents are used as models for sarcopenia since these species mimic the clinical phase of the human condition [15,31,62,74,75,76], and here, we chose rats, rather than mice, because available data suggest that the sarcopenia process in rats is more similar to the human counterpart (idem). We selected an outbred rat strain to capture the variability in emergence and progression of sarcopenia, and the results show that endpoint rats doing well had outcomes like average early-aged rats and vice versa. Thus, the variance between chronological and biological age seen here in rats resembles the situation reported in humans. Sarcopenia in humans is brought to our attention once the condition infringes on the physical activity of daily living or, for example, results in a fall-related fracture. Thus, in this study, we used motor capacity and coordination indices along with relative muscle mass as key metrics of sarcopenia. We placed the emphasis on gait performance because this entity appears to be a good biomarker for human sarcopenia (see Introduction) and translates well between species [68,74,77].

We used the soleus component of the hindlimb triceps surae muscle. The rationale for selecting this muscle was its frequent use and long duty cycle in the daily motor behaviors of a freely moving rat [67], and an ankle extensor muscle is highly relevant since rats normally stride on their toes. The drawback is that the morphological and transcriptional data on humans usually derive from the vastus lateralis component of the musculus quadriceps femoris (an extensor of the knee joint), mainly because it is readily accessible for tissue sampling. 

In this study, no net change in the number of myofibers of the soleus muscle across the adult life span could be detected. Our data on fibre numbers agree with earlier observations made in this species [72,78], and they were assessed in a complete cross-section at a distance from its origin where most if not all fibers should be present. While the loss of myofibers has been reported in the m. vastus lateralis in humans, it is also known that the sarcopenia process impacts muscles differentially, and m. soleus, serving as an antigravity postural muscle with long duty cycles, is affected at a later stage and to a lesser extent than for example the components of m. quadriceps (for review, see [79]). The incapacitation of m. soleus marks a progression stage of sarcopenia where individuals are unable to maintain an independent lifestyle. This was also the case for many subjects at the endpoint in this study being unable to reach the food hopper or nipple of the water bottle (see Material and Methods).

We used embryonic myosin (MyHC3), NCAM, and CHNR-γ in parallel as markers of myofiber denervation. MyHC3 is normally suppressed in adult muscle but re-expressed following denervation and resuppressed upon reinnervation (in the rat, [65]; reviewed in [80]) and often used as a marker for denervation in studies of aging ([68]). NCAM (neuronal cell adhesion molecule) is also frequently used as a marker for denervation ([81]; reviewed in [82]). Sanes and colleagues showed that the NCAM protein associates with the postsynaptic membrane of the NMJ ([83]) and, moreover, that disturbance of the NMJ integrity results in an upregulation of NCAM by the myofiber (idem). However, using either one of NCAM or MyHC3 as a denervation marker may underestimate the true number because these proteins are not distributed uniformly along the length of a myofiber ([81]). As a third marker for denervation, we assessed the mRNA expression of the CHNR-γ subunit. The CHNR is composed of five subunits which change in composition from α, β, δ, γ to α, β, δ, ε during development but revert back in response to denervation ([65,84]). In adult rats, the re-expression is resuppressed following successful reinnervation ([65]). As recently shown, reversible changes in the expression of the markers neonatal MyHC (MyHC8), NCAM, and CHNR-γ can also be induced by muscle unloading alone ([85,86]), an intervention that in addition induces changes to the prevalence of fast and slow myofibers and the degree of fiber-type grouping (*idem*, see above). Thus, the currently deployed markers for myofiber denervation cannot safely make a distinction between changes induced by nerve damage and unloading. Another marker used for myofiber denervation not included here is the relative expression of the voltage-gated sodium channels Nav1.4 and Nav1.5 [87,88]. However, as shown elsewhere, Nav 1.4 and Nav 1.5 are differently regulated depending on context ([89,90]) and neither Nav 1.5 nor NCAM is consistently induced by denervation in young and old rats ([82]).

The mRNA transcript analyses were conducted on whole muscle samples and can thus not provide information about which cell type(s) expressed the different mRNAs. The selection of transcripts analyzed herein was based on the results of our previous longitudinal study of muscle aging in humans [66] and the temporal pattern of transcript regulations in the response to peripheral nerve crush in female rats [65]. 

A further limitation of this study is the cross-sectional design (for discussion on study design and references, see [66]). However, we believe this is less of a problem with rats since their lifespan is comparatively short and living conditions can be kept highly reproducible across age groups.

As mentioned in the introductory paragraphs, the emergence and progression of sarcopenia are based not only on changes in the myofibers and their innervation. The muscle scaffold (i.e., the soft supporting skeleton of the muscle) exhibits significant changes during aging, and its supporting role for the contractile elements may dissolve with age [91,92,93]. Recently, the importance of cross-communication between different tissues (e.g., muscles, joints, skeleton, immune and endocrine systems) has received considerable attention, and these communication channels may have an impact on the instigation and progression of sarcopenia [91,94,95,96]. Certainly, these aspects should be considered in future studies.

### 4.2. Natural History of Sarcopenia in the Rat

#### 4.2.1. Preclinical Phase of Sarcopenia

The results show that motor performances such as gait and coordination remain intact through middle age (12–18 months) when muscle mass relative to body weight is largely maintained. The muscle, however, shows multiple signs of remodeling (adaptations) with a shift in myofiber-type composition toward type I, an increased number of hybrid fibers, and of, in particular, type II fibers expressing embryonic myosin, a pattern shift apposite to that observed following soleus muscle unloading or nerve damage in young adult rats ([65,97]). In late middle age, almost 10% of mainly type II myofibers coexpress MyHC3, and in parallel, the number of hybrid fibres rise to the same level. However, these two fiber subpopulations are not completely overlapping, implicating that some hybrid fibers have been reinnervated and do not express embryonic myosin. Also considering the parallel decrease in the number of type II fibers between adulthood (19%) and late middle age (13%), the pattern shift in late middle age is not likely induced by sustained unloading or timed nerve damage but, as suggested by Lexell and coworkers [22], by repeated cycles of denervation (of preferentially type II myofibers followed by collateral reinnervation (by motoneurons innervating type I myofibers). The increase in MyHC3-IR protein correlated with a robust upregulation of NCAM (neuronal cell adhesion molecule), nicotinic acetylcholine receptor subunit γ (CHRNγ), and MYOG mRNA. The covariation in NCAM, MYOG, and CHNR-γ mRNA upregulations observed here in aging male and female rats is similar to that occurring in response to experimental denervation in young adult rats (this study and [11]) and in line with corresponding data from aging female but not male humans [18,19].

The number of MyHC3-IR type II fibers combined with the upregulation of CHNRγ and NCAM mRNA suggests that the remodeling is driven by a denervation of type II myofibers belonging to fast-twitch MUs, followed by a collateral reinnervation from intact axons innervating nearby type I myofibers of slow-twitch MUs. That the recruitment order and firing rate of the innervating motoneurons impact the contractile properties of the target myofibers is well known since the classical cross-innervation experiments half a century ago [98]. The selective vulnerability of myofibers in fast-twitch MU has not been clarified. However, as suggested elsewhere, the vulnerability may stem from their much more extensive axonal arborization and larger number of axon terminals, than have the motoneurons of the smaller-sized slow-twitch MUs [8]. 

Repeated cycles of denervation–reinnervation may cause a shift in fibertype prevalence and a clustering (enclosure) of fibers expressing the same contractile proteins (fiber-type grouping) and expand the size of slow-twitch MU [57,73,74,75]. Jennekens and colleagues brought our attention to the fact that the mosaic pattern of myofibertype distribution within a mixed muscle differed among human skeletal muscles and, moreover, is impacted by myopathological processes including aging [35,69]. The increased fiber-type grouping observed during aging was later confirmed and extended by Lexell and coworkers [99,100]. We observed an increase from ~20% up to ~50% of enclosed type I myofibers in the soleus muscle during aging (see also [54]). In our material, the observed number of neighbors of a type I fiber came very close to the assumed model of a hexagonal lattice array arrangement of cross-sectioned myofiber profiles in all age groups and with a standard deviation of close to 1. Furthermore, ~70% of our observations on the frequency of enclosed type I fibers were within ±1 SD (p^6^ to p^8^) of that expected from the prevalence of type I fibers in the different age groups. The results obtained here agree with those recently reported for sedentary and exercised aging humans [101] and suggest that the degree, or changes in the degree, of fiber-type grouping is driven by an underlying shift in the prevalence of a myofiber type (see also [102,103]). However, there are also data from the human vastus lateralis muscle showing an increased type I clustering in the absence of any change in fiber-type prevalence [104].

In the preclinical phase of sarcopenia when the rats were behaviorally asymptomatic, we recorded a small drop in normalized soleus mass and a significant reduction in CSA (−15%) among the remaining type II myofibers. The concurrent decrease in the number and CSA of type II myofibers and the increase in the number (+8%) and small increase in CSA (+9%) of type I myofibers appear to largely balance this drop, resulting in a fairly stable relative muscle mass. It is notable that in contrast to more advanced ages, myofibers expressing MyHC3 in late middle age are not severely atrophied. This may be interpreted as the reinnervation at this stage being fairly rapid and frequently successful. This may help to explain the absence of behavioral signs in, for example, gait. 

In the preclinical phase during late middle age, we recorded a doubling in the number of nuclei with a central position of the myofibers and a robust upregulation of MYOG transcripts (member of the myogenic differentiation factor (MDF) family). In parallel, there was a slight (statistically not significant) increase in Pax7-IR nuclei in the satellite cell (SC) niche and an increase in βCatenin mRNA. These changes suggest that the SC population density and some of the proteins involved in SC replication and the subsequent differentiation to myocytes (MYOG and MYOD) are at least maintained at the level of young adult rats. The early upregulation of MYOG may also relate to the notion that the family of myogenic differentiation factors (MDFs) has been implicated in stabilizing the NMJ [105]. 

In experimental or disease-related atrophy of skeletal muscle, the activation of FOXO transcription factors by an increased Smad signaling (e.g., downstream of myostatin activation of the activin receptor 2) induces regulated proteolysis targeting contractile muscle proteins through the activation of the ubiquitin–proteasomal system (UPS), including the muscle-enriched E3 ligases FBXO32 (MAFbx, atrogin) and MuRF1 [106,107,108,109,110,111]. Our results show that Smad3, FBXO32, and MuRF1 (the latter not statistically significant) transcripts increase in skeletal muscle during late middle age, accompanying the adaptive changes during late middle age. We recently reported in a longitudinal study of a human cohort (ULSAM, males subjects) that the vastus lateralis muscle of m. quadriceps showed increased levels of Smad2 and MurF1 mRNAs in early aging (70 years old), while these upregulations were attenuated when the subjects were re-examined at 88 years of age [66]. The activation of the UPS during the preclinical phase of sarcopenia may be a prerequisite for the remodeling needed to adapt to the ongoing denervation–reinnervation process [66,112]. At early aging, these ULSAM subjects also showed upregulations of MYOG, MYOD, NCAM, and MyHC3 mRNAs (idem), very similar to the regulatory pattern found here in late middle-age and early-aging rats.

#### 4.2.2. Clinical Phase of Sarcopenia

Much more is known about the clinical than the preclinical phase of sarcopenia, and the results obtained in this study are largely in agreement with previous studies on male and female rats and humans. Briefly, in early (25-month-old) and late (endpoint, 30-month-old) aging, motor performance such as gait and limb coordination deteriorates as muscle mass is lost (in relative terms >50% at the endpoint). This is a gradual transition varying in pace and extent between subjects, e.g., some 25-month-old rats were still functionally intact, while others displayed serious behavioral deficits. In relation to the expected median lifespan, the 25-month-old cohort corresponds to humans at the age of 60- to 70-years old. At the level of the muscle, there is a reduction in both absolute and relative soleus muscle mass, frequency, and CSA of type II fibers, while type I fiber CSA is still maintained at the young adult level. The occurrence of hybrid- and MyHC3-IR myofibers remains about the same as in late middle age. A marked difference compared to rats in late middle age is the significant atrophy of MyHC II-, hybrid-, and MyHC3-IR myofibers, along with a further upregulation of CHRNγ, NCAM, and MYOG mRNAs. This may be interpreted as denervated myofibers to some extent remaining denervated, at least for a longer period allowing time for an accentuated myofiber atrophy and tissue accumulation of mRNA-markers of disturbed innervation. Another important difference is that in early (and late) aging, an increasing number of type I myofibers express embryonic myosin, indicating that this subpopulation of MUs becomes affected to a larger extent. Combined, these changes might explain why clinical symptoms emerge at this stage while relative muscle mass is still reduced by only ~20%. The progressive failure of collateral reinnervation with advancing age has also been proposed to occur in humans [55,57] when sarcopenia becomes clinically overt [21,57,113,114,115,116,117]. In early aging, the density of Pax7-IR nuclei and level βCatenin mRNA was lower than in 18-month-olds but still on par with our observations in young adult rats, and the marker we used as an indicator of the SC replication rate in vivo (Ki-67 IR; [70,118]) did not indicate a significant change across the adult lifespan (see also below). In early aging, we observed a transcriptional coupregulation of TGFβ1 and MYOD (member of the MDF family) mRNA not present in the middle ages; a coregulation of these two transcripts appears to play a role in the myogenic differentiation process [105,119], suggesting that more SC offspring differentiate into myocytes than in late middle life. However, both MYOD and MYOG have also been associated with other remodeling processes such as the myofiber response to denervation [112,120,121,122,123].

As aging progresses toward the endpoint, myofiber atrophy accelerates and also involves MyHC I-IR fibers (>50% reduction in CSA; see also [124]). Muscle mass is lost at a high pace also in relative terms (−60%), and motor performance and coordination deteriorate. We confirm previous observations that mRNA transcripts associated with the increased proteolysis of contractile proteins and MyHC3 mRNA return toward baseline values at advanced age [63,64,66,125], while the MDFs and NCAM, and CHRNγ transcripts remain elevated, and the percentage of myofibers with nuclei in a central position continues to increase. Also, in these respects, the current results in aging rats are in line with longitudinal observations made on aging males in the ULSAM cohort [66]. Our observations in rats at advanced age suggest that remodeling and regeneration fade but do not cease. At this stage, there is a significant depletion of Pax7-IR nuclei in the SC niche, while the replication rate among the remaining SCs was unchanged compared to late middle age and early aging. A depletion of SCs at advanced age confirms previous studies on small rodents and humans alike, while the maintained replication rate is at variance with earlier observations [44,45,126,127]. There are two not mutually exclusive explanations for this discrepancy. First, previous studies on replication rate were conducted on harvested SCs ex vivo and may not reproduce the replication rate in vivo considering the multitude of changes occurring in the muscle scaffold and SC niche during advanced aging. We used Ki67-IR as a proxy for SC replication, and despite its wide use in grading tumor aggressiveness by cell division propensity [70,118], it has not been validated for SCs in an aged SC niche. Furthermore, the colocalization of Pax7 and Ki67 IR proteins does not implicate by necessity that SC offspring commit to and successfully pursue a maturation into myocytes. Furthermore, we have examined rather few subjects in each age group. Thus, this issue deserves further attention.

At advanced age, we noted a marked increase in glial-derived nerve growth factor (GDNF) mRNA, which confirms our previous observations [128,129] and is in line with data from humans [130]. GDNF is a target-derived neurotrophic factor retrogradely transported to motoneurons and critical for perinatal survival for a subpopulation of spinal motoneurons [131,132], while the function of GDNF at later stages is not fully understood. In adulthood, GDNF may be important for NMJ maintenance and reinnervation since it is promptly upregulated in response to axon damage [130]. GDNF interacts also with the GRF-1α receptor and NCAM in the myofiber, and NCAM has been proposed as an interaction partner in collateral reinnervation processes [114,133]. However, our data on rats show that NCAM upregulation precedes that of GDNF. Thus, these changes appear not to be temporarily coordinated at the transcriptional level during aging. The significance of very high levels of GDNF transcripts at the endpoint remains unclear and needs further study.

At more advanced stages of aging, there are highly significant changes to the cellular composition of the skeletal muscle, with inter- and intra-myofiber infiltration of fat and low-level inflammation possibly driven by intramuscular degenerative processes and an accumulation of senescent cells and the impact of their secretome ([33,58,59]). In parallel, there is a significant accumulation of extracellular matrix and changes to the matrix components [91,134,135] and a multitude of changes to the muscle transcriptome and proteome [60]. Already, one by one and, in particular, when occurring concurrently, these changes will transform the muscle scaffold to an environment that is unlikely to facilitate and promote adaptations and the regeneration of myofibers in generic MUs of the muscle [91,136]. It is therefore very encouraging that physical exercise can improve motor behavior, also in very old people when muscle mass does not increase in response to exercise [137,138,139].

## 5. Concluding Remarks

Our data support previous observations that gait (stride) is a sensitive biomarker of sarcopenia (and aging) [15,31,68,74,77,140], correlating closely with relative muscle mass in rats. The muscle wasting during aging is initially owing to type II myofiber atrophy driven by fiber denervation followed by a collateral reinnervation creating a shift with increased type I dominance and a growing population of hybrids and myofibers coexpressing MyHC3 and type myosin II. This latter process made up for most of the type II atrophy in late middle age, so relative muscle mass decrease was limited to ~10%, and the subjects remained behaviorally intact (preclinical phase). During early aging, myofiber atrophy progresses, affecting both type II and MyHC3-expressing myofibers, suggesting delayed or even failed reinnervation. Although relative muscle mass loss still was modest (~−20%), gait and coordination were impacted (clinical phase). In the further progression into advanced age (endpoint), relative muscle mass loss is massive (relative mass down by ~50% from early aging to the endpoint), involving all myofiber types, and in parallel, motor performance deteriorates to the degree that several rats could not even manage the motor activity of everyday life (food and drink had to be served at the level of the cage floor). Our data suggest that rats can serve as a useful model of preclinical sarcopenia in humans. Interventions supporting the intrinsic adaptive mechanisms in operation during middle age and impeding further progression into clinical sarcopenia may prove more rewarding than addressing this condition once it has become clinically manifest. Gait (and similar motor-dependent behaviors), relative muscle mass, the expression of embryonic myosin, and the expression level of NCAM and CHRNγ appear to be useful biomarkers of sarcopenia progression in rats and humans alike.

## Figures and Tables

**Table 1:** Study population.

Cohort	Sex	Age (Months)	Strain	Vendor	Cage Type	Temp °C	Humidity (%)	Feed	n
M5m	Male	5	CR-SD	CR	Type 4 Open	21 ± 2	40–60	Lactamin R70	10
M12m	Male	12	CR-SD	CR	Type 4 Open	21 ± 2	40–60	Lactamin R70	9
M18m	Male	18	CR-SD	CR	Type 4 Open	21 ± 2	40–60	Lactamin R70	11
M25m	Male	25	CR-SD	CR	Type 4 Open	21 ± 2	40–60	Lactamin R70	8
M30m	Male	30	Sprague Dawley	Scanbur	Type 4 Open	21 ± 2	40–60	Lactamin R70	16
F3m&F3mA	Female	3	Sprague Dawley	Scanbur	Type 4 Open	21 ± 2	40–60	Lactamin R70	30
F12m	Female	12	Sprague Dawley	Scanbur	Type 4 Open	21 ± 2	40–60	Lactamin R70	10
F30m	Female	30	Sprague Dawley	Scanbur	Type 4 Open	21 ± 2	40–60	Lactamin R70	10
Total	Males								55
Total	Females								50

**Table 2:** Behavioral tests and gross measures.

			Behavioral Tests					Gross Measures		
Cohort	Sex	Age (Months)	OF	Beam Balance	Mesh Crossing	Gait	Gait-Stage	BW	m. sol Weight	SI
M5m	Male	5	X	X	X	X	X	X	X	X
M12m	Male	12	X	X	X	X	X	X	X	X
M18m	Male	18	ND	ND	ND	ND	X	X	X	X
M25m	Male	25	X	X	X	ND	X	X	X	X
M30m	Male	30	X	X	X	X	X	X	X	X
F3m	Female	3	X	X	X	X	X	X *	X *	X *
F12m	Female	12	X	X	X	X	X	X *	X *	X *
F30m	Female	30	X	X	X	X	X	X *	X *	X *

* data replotted from [64].

## Data Availability

All data files used for analysis will be made available on request to the corresponding author.

## Supplementary Materials

The following supporting information can be downloaded at: https://www.mdpi.com/article/10.3390/biology12091177/s1, Protocols for microscopy and stereology including Figures S1 and S2: Protocol for tissue staining and immunohistochemistry, including list of primary and secondary antibodies used, Table S1: Protocol for RNA extraction, qPCR and primers Table S2: used in this study; Figure S3: age-group wise comparison of gross measures; Figure S4: shows PCA analysis of the outcome from the behavioral test battery including early-aged male rat; Figure S5: age-group wise comparison of the behavioral results within sexes; Figure S6: showing the full cross-section of a soleus muscle of a young adult and an advanced aged rat; Table S3: summarizing the myofiber data (mean ±SD) for each age group of the male rats; Table S4: summarizing the data on central nuclei and satellite cells in the male rat soleus muscle; Figure S7: Boxplot of the number of myofibers in m soleus in each of the different age groups studied; Figure S8: Boxplots showing the response to sciatic nerve transection on the expression level of CHRNγ, NCAM, and GDNF mRNAs in young adult female rats [141,142].
