# Supplementary material for: Emergence and Progression of Behavioral Motor Deficits and Skeletal Muscle Atrophy across the Adult Lifespan of the Rat
Source: Biology (Basel). 2023 Aug 28;12(9):1177. doi: 10.3390/biology12091177 (PMC10526071; doi:10.3390/biology12091177)
Supplement: Supplementary file 1 [file biology-12-01177-s001.zip › biology-2520057-supplementary.pdf]

Supplementary Materials

Emergence and Progression of Behavioral Motor Deficits and Skeletal Muscle Atrophy across the Adult Lifespan of the Rat

Max GrönholdtKlein, Ali Gorzi, Lingzhan Wang, Erik Edström, Eric Rullman, Mikael Altun and Brun Ulfhake

Details of methods and chemicals used in this study according to previously published protocols [65,68,29,141,142].

Microscopy and Stereology

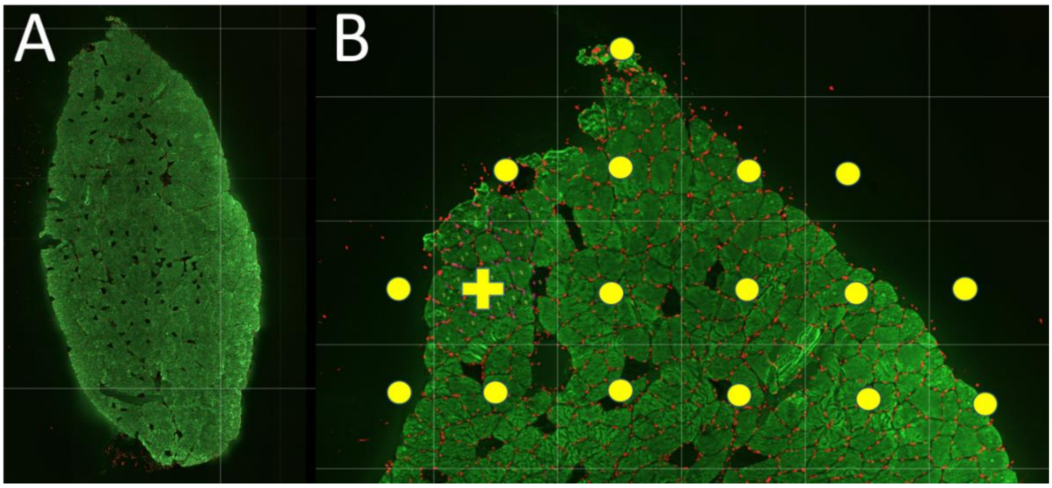

Figure S1. Photomontage of confocal microscopic images covering the full cross-sectional area of m. soleus labelled för MyHC I **(A)**. In **(B)** blow-up of the top part of the cross-section in **(A)**. Overlay (in yellow) is a grid where each square corresponds to 300 kpixles to enable unbiased systematic sampling **(B)**. The squares are counted (yellow dots) starting from the top, reading left to right (numbers). First square is generated from random numbers (1-10) and is in this example 7 (yellow cross) and then every 15th subsequent square is used until 200 fibers have been collected. Fibers completely inside the grid box or touching/stretching outside the top or left borders were included while fiber not confined inside of the right or lower borders of the grid box were excluded.

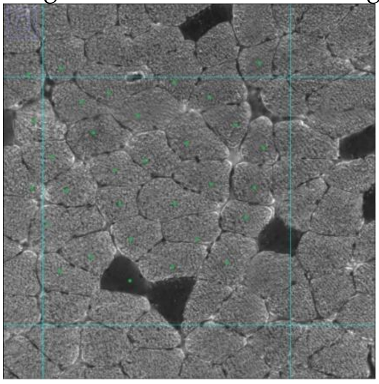

Figure S2. Close up micrograph of a section through m. soleus stained with an antibody towards MyHC 1(slow). Superimposed is the grid (green lines) subdividing the cross sections into grid boxes

used for an unbiased sampling of fibers to be included in the count. Fibers completely inside the grid box or touching/stretching outside the top or left borders were included (green dots) while fiber not confined inside of the right or lower borders of the grid box were excluded.

Hematoxylin and eosin staining protocol.

1. Air dry slides at room temperature.
2. Rehydrate tissue with 0.1M PBS (phosphate-buffered saline) for 1-2 minutes.
3. Immerse slides in Mayer's hematoxylin for 5 minutes.
4. Rinse slides in ordinary water for 3x10 minutes on Stovall Belly dancer 4702610 with two changes of water in between.
5. Immerse slides in eosin 0.2% for 8 minutes
6. Immerse slides in 75% and 95% ethanol for 2x1 minutes respectively.
7. Mount slides with 2.5% DABCO in glycerol.

Immunohistochemistry protocol

Tissues were dried in RT for 30 min. A well was created using a DAKO pen. The tissue was then fixated in 4% formaldehyde for 10 minutes then rinsed in cold tap water for 10 minutes repeated three times. Blocking was performed with 5% normal donkey serum for one hour in RT. Primary antibody was incubated for either 1h at 37 ^{o}C or overnight at 4 ^{o}C. In case of dual epitope labeling, this step was repeated following washing. After primary antibody incubation, the washing sequence above was repeated. Secondary antibody was incubated for one hour at RT covered from light. The samples were mounted with DAPI-antifade (Vectashield, H-1200) and left to incubate covered from exposure to light for 20 min at RT before imaging or storage in 8-12 ^{o}C. DAPI served as unspecific nuclear stain in microscopical multicolor analyses.

Table S1. Primary and secondary antibodies used.

Source	Species	Type of ab	Dilution
sMHC (MyHC I)	Mo	Novocastra	1:10
fMHC (MyHC II)	Mo	Novocastra	1:50
BF-G6(MyHC 3)	Mo	Developmental Studies Hybridoma Bank, Ohio 2011	3μg/ml
MyHC 3	Mo	Novocastra	1:50
Laminin PC128	Sheep	The Binding Site	0.7-1ug/ml
Laminin L9393	Rb	Sigma	1:800
Pax7	Mo	DS Hybridoma Bank	1-3 ug/ml
Ki67	Rb	Novocastra	1:1000

RNA extraction, cyber-green PCR, and primers

12-24mg of tissue
⅓ of 2ml vial filled with zirconia beads
1ml Trizol to each screw top vial
Add biopsy
Homogenate 15sec/round, check that all samples dissolved, if still solid tissue re-run only that vial
Incubate 5min at RT
Add 200ul of chloroform to each vial
Shake 15 sec, all at once kept in the rack
Incubate 3 min at RT (when doing several samples incubate on ice until all samples are processed → initial phase separation starts)
Spin at 12000g 15 min at 4 ºC (samples separated into upper RNA and lower DNA+protein layer)
Collect upper aqueous phase and place in RNA-vial. Do not disturb the interphase!
Add 500ul of isopropanol (2-propanol) to the RNA-phase → immediately invert tube manually and vortex full speed (about 30 sec)
Incubate at RT for 10 min
Spin at 12000g for 10 min at 4 ºC
Prepare 75% ethanol (dilute 100% EtOH with milliQwater → pre-cool on ice
Gently pour off the liquid (in hazard wastes in the hood), careful not to lose the pellet. The last part of fluid is removed with 100ul pipette
Add 1 ml (ice-cold) 75% ethanol to the pellet and wash by vortexing gently (the pellet should loosen from the bottom of tube)
Spin at 7500g for 10 min at 4 ºC
Discard supernatant, remove the last amount of liquid using pipette.
Semi-dry the pellet 5 min at RT
add 20ul MilliQ water on top of the RNA pellet
Leave at RT for 5-10 min
Vortex carefully for about 1 min to dissolve pellet. Quick spin if necessary to collect drops on walls
Store at -80 ºC

Concentration measurement

Was done with PicoDrop; Picopet01, Picodrop Ltd, Cambridge UK

RNA integrity

RNA integrity was measured with Experion™ RNA StdSens & HighSens Analysis Kits

Reverse Transcription

Applied Biosystems High-Capacity cDNA Reverse Transcription Kit. 300ng RNA in a 20ul reaction volume diluted 1:20 for a final calculated cDNA-concentration of 0,75ng/µl.

Incubate at 25 ºC for 10 min
Transcription 2h at 37 ºC
Inactivate at 85 ºC for 5 s

PCR

Enzymes Dynamo Colorflash SYBR Green qPCR Kit
Primer (20uM) U+L 0,5ul / well
cDNA (0,75ng/µl) 2,5ul / well
2X Mastermix 6,25µl / well
Nucleotide free H2O 3,25ul / well
Reaction volume 12,5 µl
Holding stage 7 min 95 ºC
Cycling stage 10s 95 ºC, 30s 60C X40
Melting Curve 15s 95 ºC, 1 min 60 C, slow incremental heating up to 95 C

Table S2. Primers used in qPCR

Gene	Accession number	Forward primer	Revers primer
GPx1	NM_030826	CCGGGACTACACCGAAATGA	CTGATGTCCGAACTGATTGCA
β Actin			
AChR◎◎(CHRNγ)	NM_009604	GATGCAATGGTGCGACTATCGC	GCCTCCGGGTCAATGAAGATCC
MuRF1 (TRIM63)	NM_080903.1	TGTCTGGAGGTCGTTTCCG	ATGCCGGTCCATGATCACTT
Atrogin (MAFbx/FBXO32)	NM_133521	CCATCAGGAGAAGTGGATCTATGT	GCTTCCCCCAAAGTGCAGTA
Smad3	NM_013095	CATCTACTGCCGCTTGTGG	TCATGTTGAAGGCGAACTCAC
Myogenin (MYOG)	NM_017115.2	ACCATGCCCAACTGAGATT	TGGGCAGGGTGTTAGTCTT
MyoD (MYF3, MYOD1)	M84176	GACGCCGCCTACTACAGTGAG	CCACCCCTCAGTGTCCTGCAGG
TGF-◎◎	NM_021578	CTGAACCAAGGAGACGGAATAC	CCAGGTCACCTCGACGTT
◎-Catenin (CTNNB1)	NM_053357	TCACCACGCTGCATAATC	ATAGCCAAGAACTTCACGTTT
Glial-derived neurotrophic factor (GDNF)	NM_012528	AAATCGGGGGTGCGTCTT	ATGCCTGGCCTACCTTGTCA
Neural cell adhesion molecule (NCAM, CD56)	NM_031521	CAGCGGATCTCAGTGGTGTG	TCTCGGCCTTTGTGTTTCCAG

Confocal microscopy

Microscopy and Image Processing. Representative images were acquired from one airy unit pinhole on an LSM700 confocal laser scanning microscope (Carl Zeiss) equipped with an objective with a magnification of x5, an EC Plan-Neofluar objective with a magnification of x10 and N.A. of 0.30, a Plan-Apochromat M27 objective with a magnification of x20 and N.A. of 0.80. Emission spectra for each dye were filtered as follows: FITC (505–540 nm), Cy3 (560–610 nm), and Cy5 (>640 nm). Images were processed using ZEN2012 software (Zeiss). Multi panel figures were assembled using Adobe Photoshop CS6 software (Adobe Systems).

Fiber type imaging was done according to Zhang et al 2014 adjusted for our circumstances. We thank Dr Jan Mulder for generously allowing us to use the equipment.

Supportive information of Results

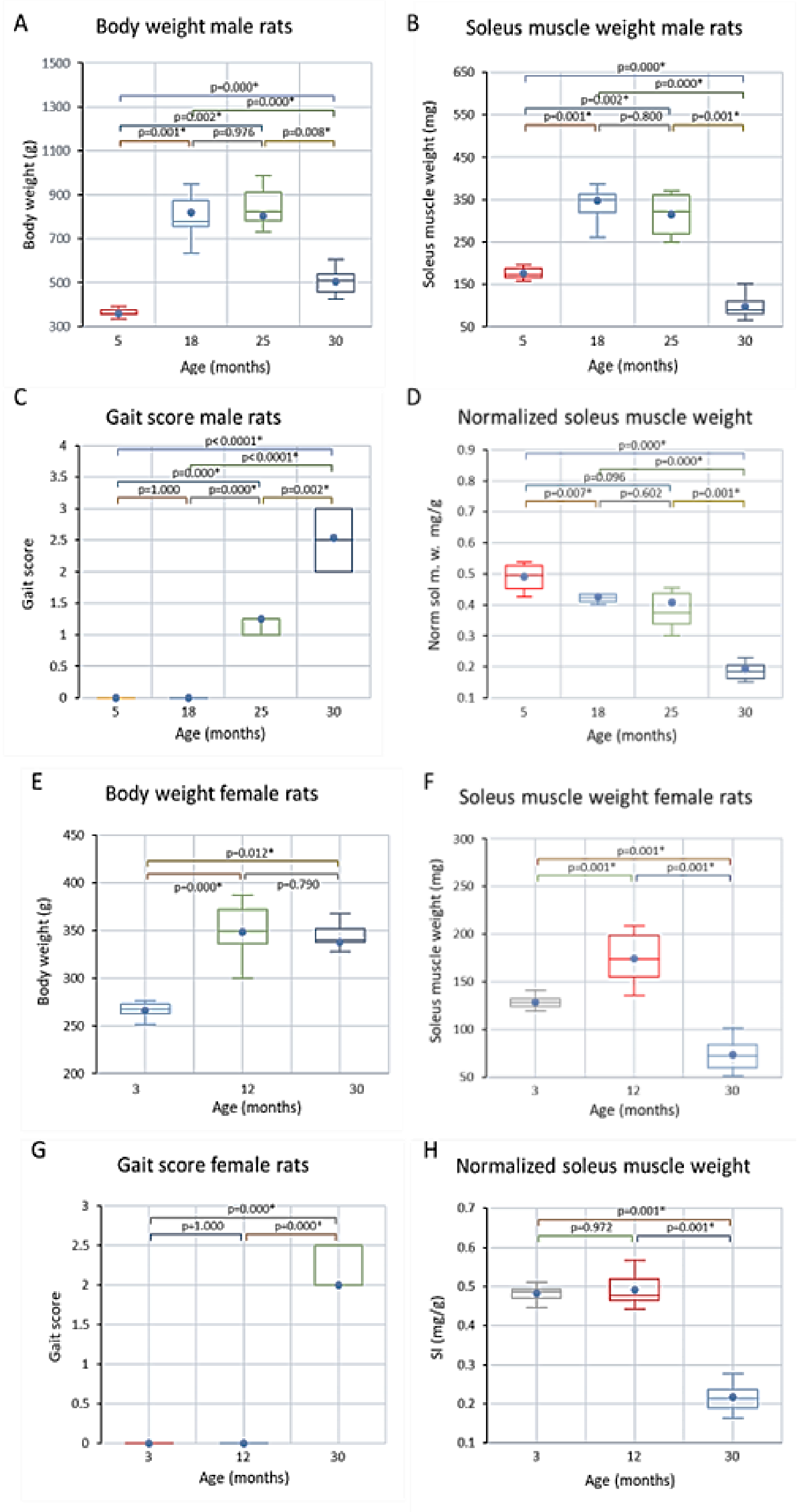

Figure S3. Boxplots of male (A-D) and female (E-H) rats whole body weight (A, E), m. soleus muscle weight (B, F), gait score (C, G) and soleus muscle weight normalized to body weight (D, H) across adult lifespan. The variation across lifespan was significant for each parameter in both sexes (KW <0.001). In the plots, the outcome of pairwise post hoc testing has been indicated.

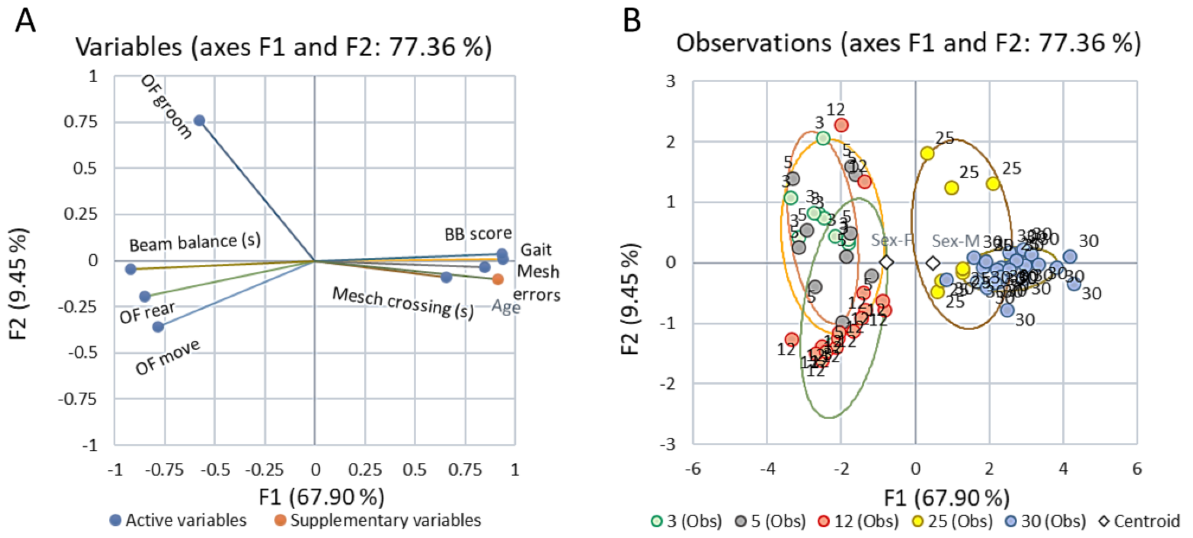

Figure S4. PCA analysis of (A) activity, motor, and coordination indicis in (B) male and female age cohorts (3-, 5-, 12- and 30-month-old) when also observations on early aged males (25-month-old; yellow markers) were included. The 25-month-old males formed a group of observations positioned in between middle aged and 30-month-old rats. Ovals indicate the 70% confidence limit of each age group.

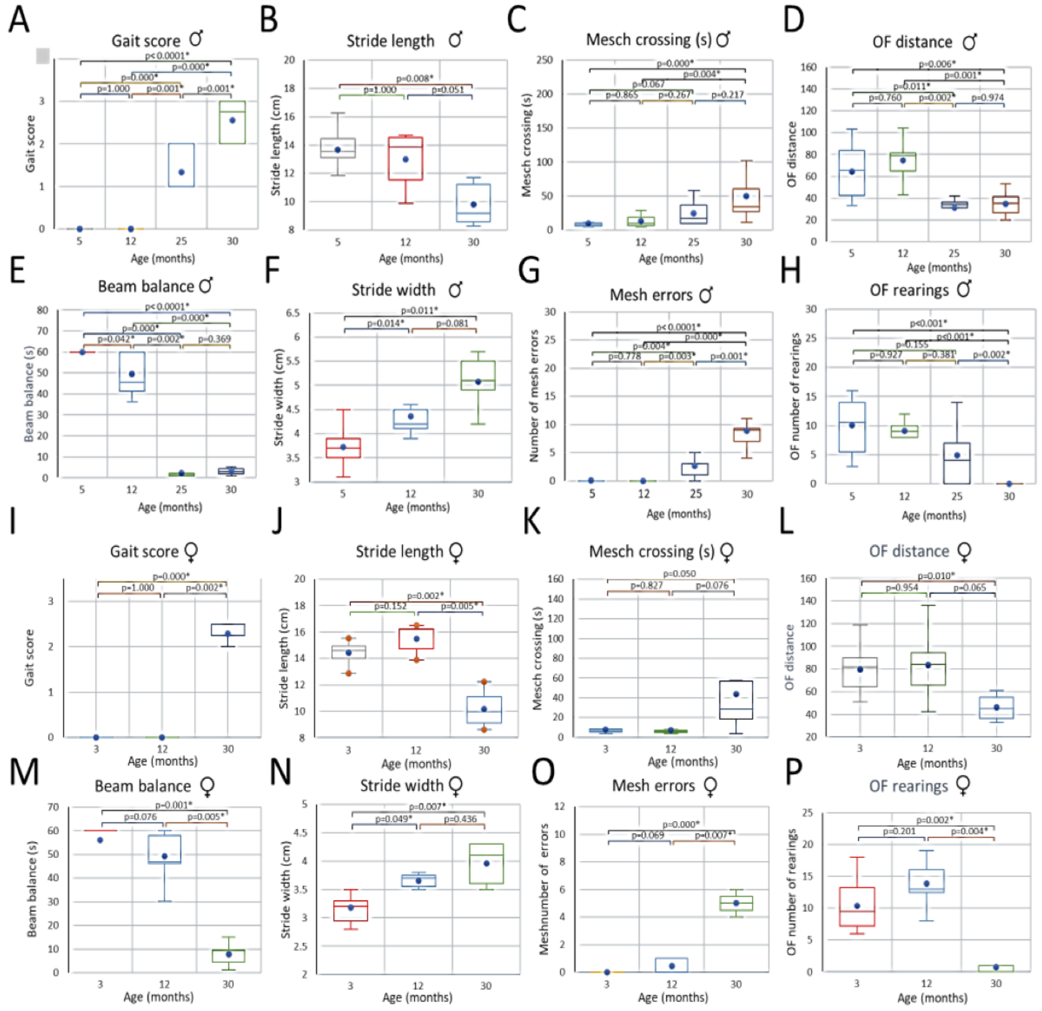

Figure S5. A-G (males) and I-P (females) show boxplots of the behavioral variables assessed across adult life span. In each panel the outcomes of the post hoc testing have been indicated.

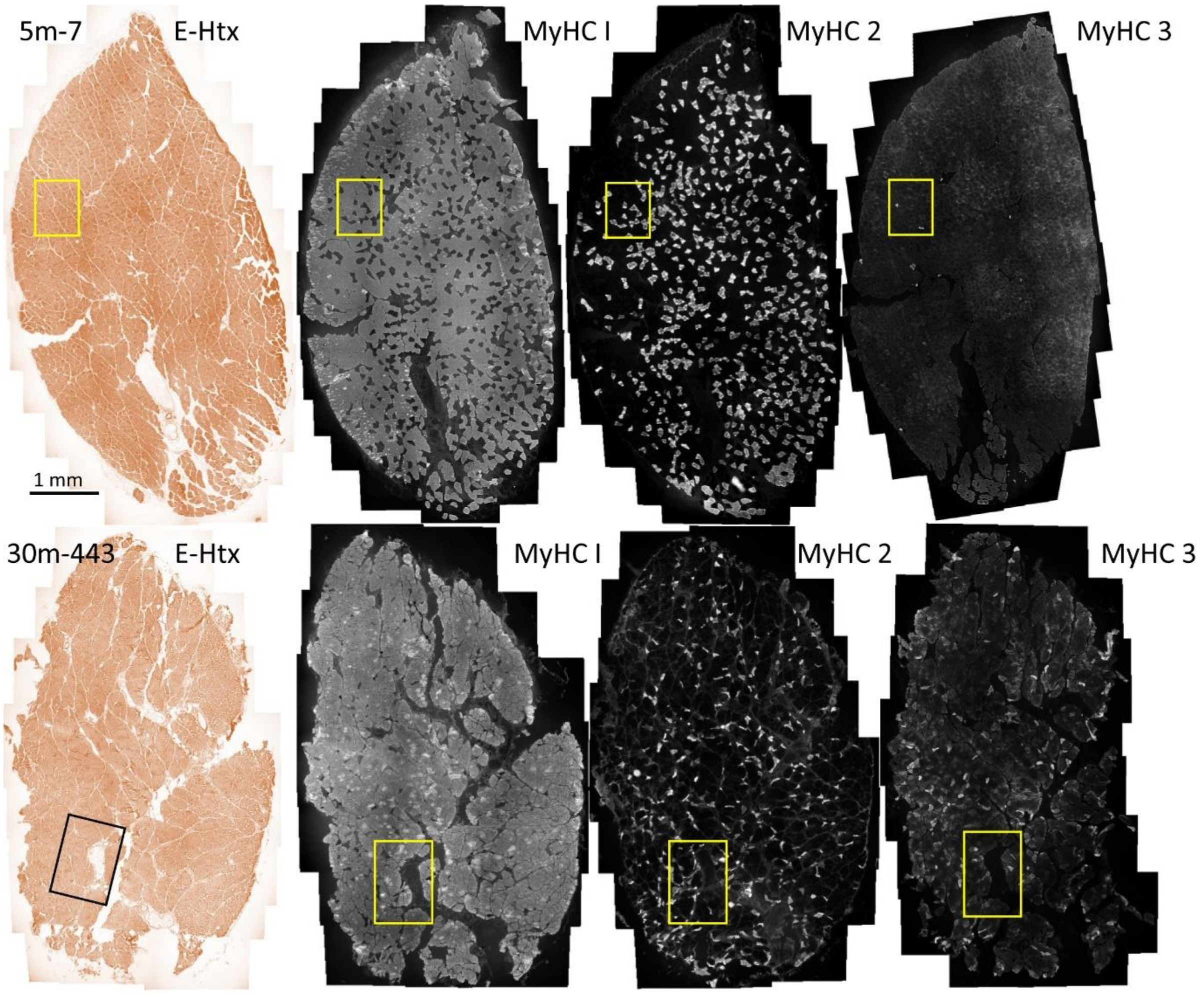

Figure S6. Photomontages of adjacent sections of the full cross section of m soleus from a young adult male rat (top panels) and a male rat at study endpoint (30-month-old) in the lower row of panels. Panels left-to-right of each row have been labelled with EosinHTX. MyHC IR, MyHC II IR, and MyHC 3 IR. Framed area in the panels show region depicted at higher magnification in Fig. 6. Magnification bar in upper left panel is valid for all panels of Fig S6.

Table S3. Gross and myofiber measures of male rats

Cohort		Age (months)	Muscle wet weight* (mg)	Norm. muscle w. (mg/g)	Gait (score)	Number of muscle fibers	Freq (%) MyHC I fibers	Freq (%) MyHC II fibers	Freq (%) hybdrid fibers	Freq (%) MyHC 3 fibers	CSA MyHC I fibers (μm^2)	CSA MyHC II fibers (μm2)	CSA MyHC III fibers (μm2)
**M5m**	mean	5	238.2	0.47	0.0	2617	80.9	19.2	0.5	0.4	4170	3626	1023
	±SD		30.7	0.02		405	4.3	4.2	0.5	0.4	931	811	859
**M18m**	mean	18	358.0	0.44	0.0	2385	87.6	13.2	8.8	8.1	4504	2861	2609
	±SD		73.4	0.02		289	3.0	2.7	3.0	3.1	1577	2053	1876
**M25m**	mean	25	329.0	0.44	1.20	2329	89.5	8.0	7.4	9.0	4251	2508	1320
	±SD		47.3	0.13	0.45	180	8.3	6.2	4.0	4.8	1865	3097	2047
**M30m**	mean	30	117.2	0.23	2.43	2843	89.1	12.9	13.4	7.4	1651	663	673
	±SD		27.4	0.04	0.53	520	5.2	5.2	4.4	2.5	1783	614	728
K-W	p=		0.001	0.002	<0.001	0.295	0.085	0.029	0.004	0.011	<0.001	<0.001	<0.001

* animals subjected to full histological analyses only

Table S4. Central nuclei and SCs

Cohort		Age	Central nuclei (%)	Pax7 IR profiles / myo-profile*	% Ki67-IR of Pax7-IR*
**M5m**	mean	5	3.0	0.15	6.54
**n=5**	±SD		0.6	0.03	4.67
**M18m**	mean	18	8.1	0.24	3.08
**n=5**	±SD		3.2	0.09	4.21
**M25m**	mean	25	6.6	0.14	2.65
**n=5**	±SD		2.4	0.05	2.52
**M30m**	mean	30	16.0	0.05	3.94
**n=5**	±SD		7.6	0.02	4.28
KW	*p=*		*0.007*	*0.005*	*0.51*

** average # fibres ≥117 analysed*

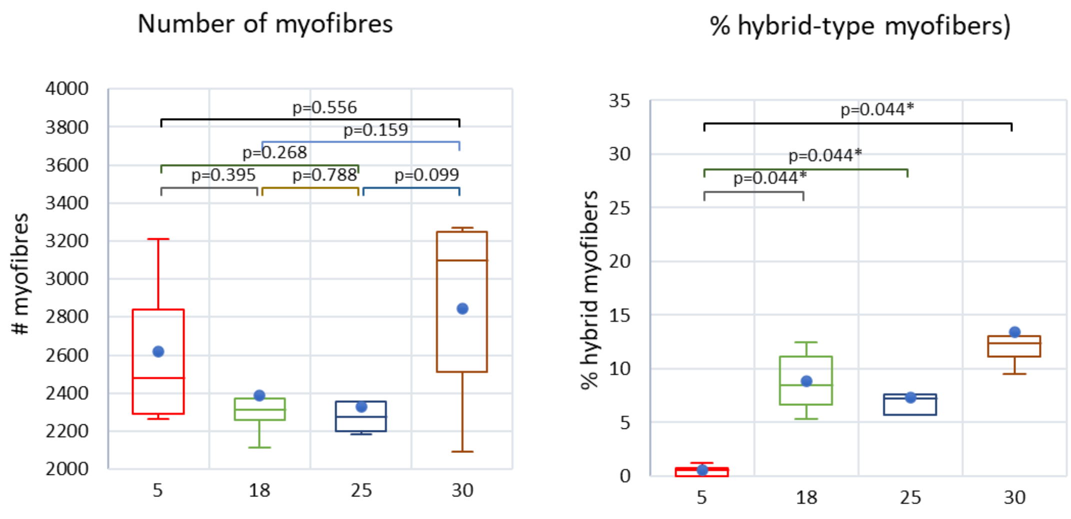

Figure S7. Boxplots of metrics (A-B) on male m soleus during aging. Age groups have been indicated on the abscissa and metric on the ordinate. In A-B the pairwise post hoc testing results have been indicated.

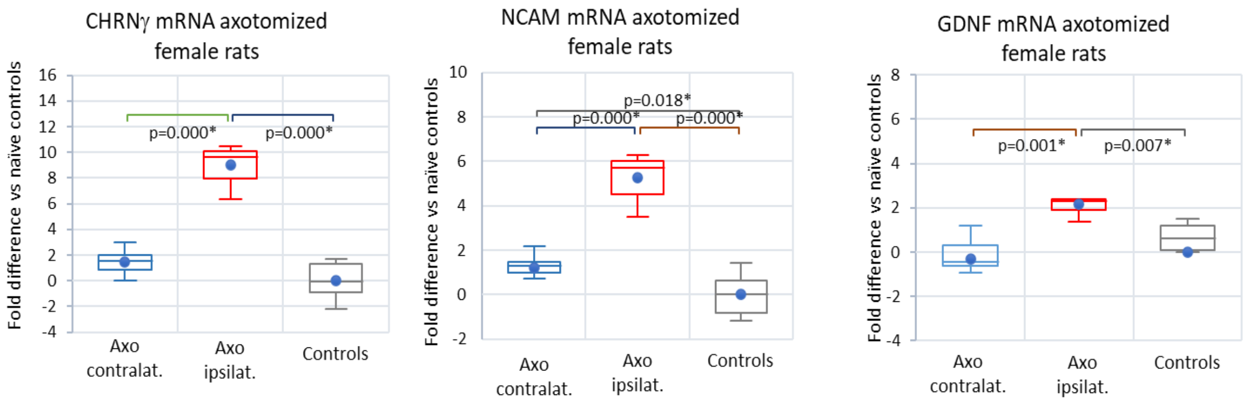

Figure S8. Boxplots of CHRNγ (left), NCAM (middle), and GDNF (right) mRNAs in the muscle response ten days after a complete unilateral axotomy in young adult female rats. In panels left-to-right the transcript level on the ipsilateral and contralateral side is shown along with naïve controls (used for normalization). Kruskal-Wallis analysis of variance indicated that each metric changed in significantly in response to axotomy. Pairwise post hoc testing results have been indicated where they were significant.

Cited literature

[65] M. Grönholdt-Klein, M. Altun, M. Becklén, E. Dickman Kahm, A. Fahlström, E. Rullman, and B. Ulfhake, Muscle atrophy and regeneration associated with behavioural loss and recovery of function after sciatic nerve crush. Acta Physiol (Oxf) 227 (2019) e13335.

[68] M. Altun, E. Bergman, E. Edstrom, H. Johnson, and B. Ulfhake, Behavioral impairments of the aging rat. Physiol Behav 92 (2007) 911-23.

[29] E. Edström, and B. Ulfhake, Sarcopenia is not due to lack of regenerative drive in senescent skeletal muscle. Aging Cell 4 (2005) 65-77.

[141] T.J. Shi, T. Tandrup, E. Bergman, Z.Q. Xu, B. Ulfhake, and T. Hokfelt, Effect of peripheral nerve injury on dorsal root ganglion neurons in the C57 BL/6J mouse: marked changes both in cell numbers and neuropeptide expression. Neuroscience 105 (2001) 249-63.

[142] M.D. Zhang, G. Tortoriello, B. Hsueh, R. Tomer, L. Ye, N. Mitsios, L. Borgius, G. Grant, O. Kiehn, M. Watanabe, M. Uhlen, J. Mulder, K. Deisseroth, T. Harkany, and T.G. Hokfelt, Neuronal calcium-binding proteins 1/2 localize to dorsal root ganglia and excitatory spinal neurons and are regulated by nerve injury. Proc Natl Acad Sci U S A 111 (2014) E1149-58.
